# Class II resin composite restorations—tunnel vs. box-only in vitro and in vivo

**DOI:** 10.1007/s00784-020-03649-y

**Published:** 2020-11-09

**Authors:** Peter J. Preusse, Julia Winter, Stefanie Amend, Matthias J. Roggendorf, Marie-Christine Dudek, Norbert Krämer, Roland Frankenberger

**Affiliations:** 1 Marburg, Germany; 2 Department of Operative Dentistry, Endodontics, and Pediatric Dentistry Medical Center for Dentistry, University Medical Center Giessen and Marburg, Campus Marburg, Georg-Voigt-Str. 3, 35039 Marburg, Germany; 3 Department of Pediatric Dentistry, Medical Center for Dentistry, University Medical Center Giessen and Marburg, Campus Giessen, Schlangenzahl 14, 35392 Giessen, Germany

**Keywords:** Resin composites, In vitro, Clinical observation, Marginal integrity, Tunnel preparation, Box-only preparation, Thermomechanical loading, In vitro vs. in vivo

## Abstract

**Purpose:**

In a combined in vitro/in vivo approach, tunnel vs. box-only resin composite restorations should be evaluated using thermomechanical loading (TML) in vitro and a restrospective clinical trial in vivo.

**Materials and methods:**

For the in vitro part, box-only and tunnel cavities were prepared in 32 extracted human third molars under simulated intraoral conditions in a phantom head. Specimens were randomly assigned to four groups (*n* = 8; 16 box-only/16 tunnel) and received bonded resin composite restorations with Amelogen Plus (box A/tunnel A) or lining with Ultraseal and Amelogen plus (box B/tunnel B) both bonded using PQ1 (all Ultradent). Specimens were subjected to a standardized aging protocol, 1-year water storage (WS) followed by TML (100,000 × 50 N; 2500 × + 5/+ 55 °C). Initially and after aging, marginal qualities were evaluated using replicas at × 200 magnification (SEM). For the corresponding in vivo observational study, 229 patients received 673 proximal resin composite restorations. From 371 tunnel restorations, 205 cavities were filled without flowable lining (tunnel A), and 166 tunnels were restored using UltraSeal as lining (tunnel B). A total of 302 teeth received conventional box-only fillings. Restorations were examined according to modified USPHS criteria during routine recalls up to 5 years of clinical service.

**Results:**

In vitro, all initial results showed 100% gap-free margins when a flowable lining was used. Tunnels without lining exhibited some proximal shortcomings already before TML and even more pronounced after TML (*p* < 0.05). After TML, percentages of gap-free margins dropped to 87–90% in enamel with lining and 70–79% without lining (*p* < 0.05). In vivo, annual failure rates for box-only were 2.2%, for tunnel A 6.1%, and for tunnel B 1.8%, respectively (*p* < 0.05). Tunnels had significantly more sufficient proximal contact points than box-only restorations (*p* < 0.05). Flowable lining was highly beneficial for clinical outcome of tunnel-restorations (*p* < 0.05).

**Conclusions:**

With a flowable lining, tunnel restorations proved to be a good alternative to box-only resin composite restorations.

**Clinical relevance:**

Class II tunnel restorations showed to be a viable alternative for box-only restorations, however, only when flowable resin composite was used as adaptation promotor for areas being difficult to access.

## Introduction

Resin composite restorations are the predominant treatment option for cavitated carious lesions because they allow for minimally invasive caries therapy being additionally esthetic and finally quite repairable [1–4]. Whereas biocompatibility of resin-based composites is per se not estimated better compared to amalgam [5], inadequate handing involves the probably highest biological risk for the patient [6]. Therefore, technique sensitivity and handling have gained significant importance from the clinical point of view [4, 6], and simplified bulk-fill materials have been more and more popular [7].

For biomaterials shrinking due to polymerization, durable adhesion to tooth hard tissues still is a fundamental prerequisite for marginal quality and therefore clinical success [7–11]. Adhesive failures result in gap formation and subsequently secondary caries which again corroborate clinical outcome [6–11]. Although bonding to enamel is still considered more effective and durable than dentin adhesion, several clinical trials already demonstrated appropriate dentin sealing and acceptably low postoperative hypersensitivities [12, 13]. Many so-called innovative materials have been developed during the last two decades such as hybrid resin composites, fine hybrid resin composites, nanohybrid resin composites, purely nano-filled resin composites, and silorane-based composites; however, using them clinically showed that general clinical problems remained similar and were more often related to handling and operator issues than material aspects alone [14].

Proximal adhesive restorative techniques until now still suffer one shortcoming: Due to the linear axis of bur rotation, access to undermining caries still requires substantial sacrifice of sound hard tissues, and in most of the restored proximal lesions, more sound than infected tissue may be removed when conventional cavity designs are cut [15–17]. Therefore, tunnel restorations have been repeatedly discussed as possible alternative to conventional box-only fillings [18–30]. It is assumed to be advantageous that tunnel preps are less invasive and may make it easier to achieve a tight proximal contact [23–26]. On the other hand, both excavation and preparation are demanding to the operating dentist, and the risk to overlooked caries as well as fracturing lateral ridges is omnipresent in scientific literature of the field [24, 25, 27–30]. However, the predominant number of papers deals with glass ionomer cements where no adhesive stabilization is provided and fractures may be logical [31–33]. Also, the routine use of flowable composites to get easier access to undermining areas is not considered in most of the clinical studies [23].

Thus, the aim of this clinical trial was to investigate two different restorative procedures (i.e., with and without flowable lining) in minimally invasive Class II cavities in vitro and in vivo, the latter with a specially designed mushroom bur for excavation. The null hypothesis tested was that there would be no difference between the different approaches (i.e., tunnel vs. box-only preparation) and applications (with and without flowable lining) in terms of marginal quality in vitro and in vivo, and to investigate the suitability of the mushroom-shaped prototype bur.

## Materials and methods

Teeth for in vitro research were extracted due to medical reasons with written informed consent of the patients. For both in vitro and in vivo investigations, approval by a local ethics committee was given (Ref. No. 143/09).

### In vitro study

**Fig. 1:**
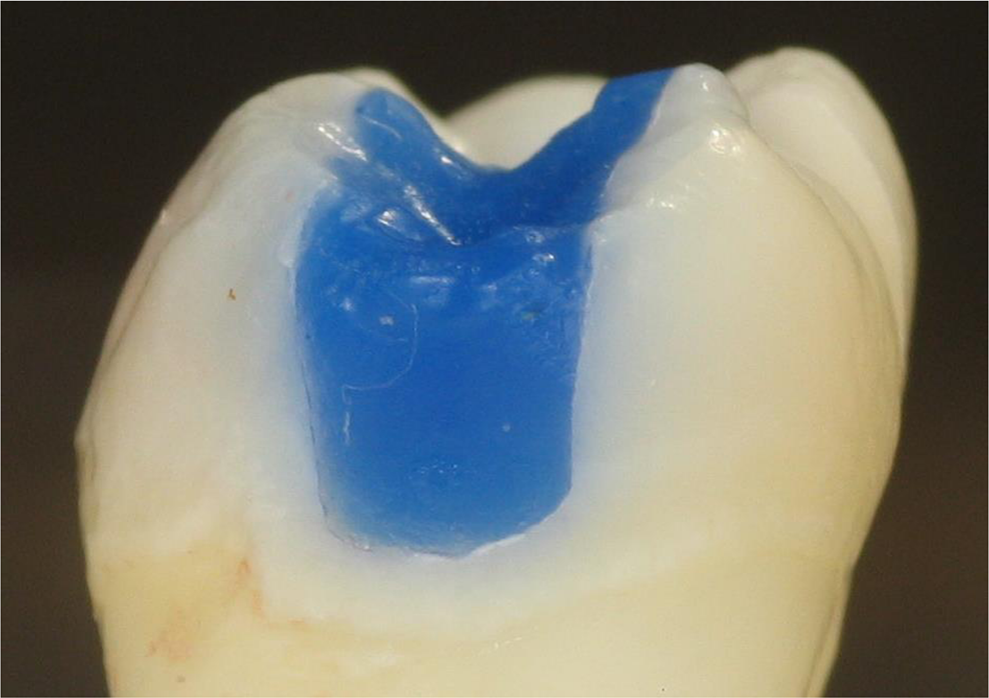
Box-only preparation in vitro

**Fig. 2:**
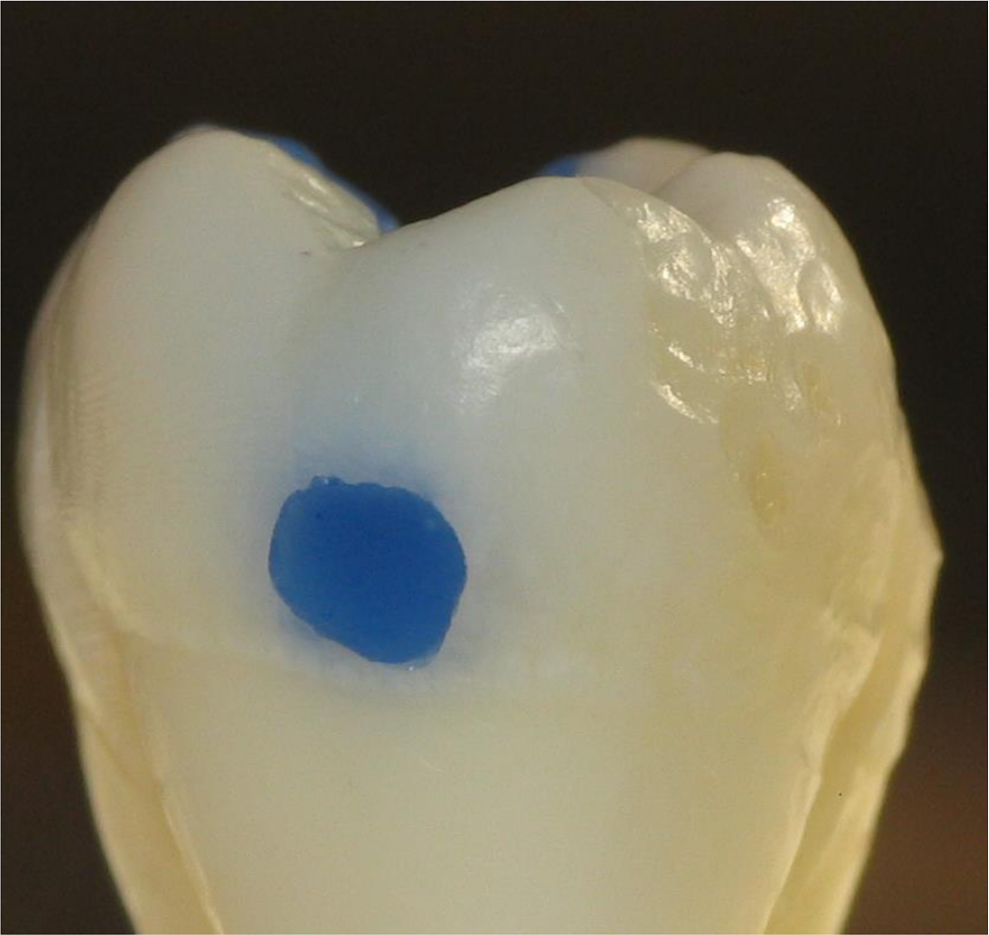
In vitro cavity with tunnel preparation, filled with blue wax for better visibility

Thirty-two intact, non-carious, unrestored human third molars, extracted for therapeutic reasons with patients’ approval, were stored in an aqueous solution of 0.5% chloramine T at 4 °C for up to 30 days. The teeth were debrided of residual plaque and calculus, and examined to ensure that they were free of defects under a light microscope at × 20 magnification. In a full-arch phantom head, standardized Class II cavity preparations (16 MO box-only, 3 mm in width bucco-lingually, 2 mm in depth at the bottom of the proximal box, completely surrounded by enamel; Fig. 1) and 16 tunnel preparations leaving the lateral ridge intact, Fig. 2) were cut. The sample size for the different in vitro groups was not decided based on statistical assumptions; it was based on experience from the discussed prior studies in the field [9–11].

The cavities were prepared using coarse diamond burs under profuse water cooling (80 μm diamond, Komet, Lemgo, Germany), and finished with a 25 μm finishing diamond (one pair of diamonds per four cavities). Inner angles of the cavities were rounded and the margins were not beveled to deliver comparable results to previous experiments and due to impaired beveling of margins in the tunnel groups. The prepared cavities (*n* = 8) were mount in a phantom head in proximal contact with two adjacent teeth. Specimens were treated with a two-step etch-and-rinse adhesive (PQ1, Ultradent, South Jordan, UT, USA; Table 1). The dentin adhesives and resin composite were polymerized with a light-curing unit (Bluephase, Ivoclar Vivadent, Schaan, Principality of Liechtenstein). The intensity of the light was checked periodically with a radiometer (Demetron, Research Corp., Danbury, CT, USA) to ensure that 1000 mW/cm^2^ was always delivered during the experiments. The adhesive was polymerized for 20 s prior to application of the resin composite in all cases. The resin composite (Amelogen, Ultradent) was used either alone (groups tunnel A and box-only A) or with a thin (< 0.5 mm) lining using a flowable resin composite (UltraSeal^b^, groups tunnel B and box-only B). Each cavity was restored incrementally with the resin composite in layers up to 2-mm thickness. The increments were separately light-cured for 40 s each with the light source in contact with the edge of the cavity. Prior to the finishing process, visible overhangs were removed using a posterior scaler (A8 S204S, Hu Friedy, Leimen, Germany). Margins were finished with flexible disks (SofLex Pop-on, 3 M Oral Care, Seefeld, Germany).

**Table 1:** Composition of used adhesive

Adhesive	Composition	Treatment
PQ1 (Ultradent, South Jordan, UT, USA)	Conditioner: 35% phosphoric acid Primer/adhesive: Canadian balsam (tree sap), 15% HEMA, TEGDMA, 40% filler with fluoride, ethanol camphorquinone, phosphate monomer	Conditioner: 15 s etch, rinse, dry gently. Primer/adhesive: 20 s agitate, air blow, light cure for 20 s.

After storage in distilled water at 37 °C for 21 days, impressions (Provil Novo, Kulzer, Hanua, Germany) of the teeth were taken and a first set of epoxy resin replicas (Alpha Die, Schütz Dental, Rosbach, Germany) was made for SEM evaluation. All groups were subjected to storage in aqua dest. at 37 °C for 365 days. After storage, thermo-mechanical loading of specimens was performed in an artificial oral environment (CS4 professional line, SD Mechatronic, Munich, Germany). Two specimens were arranged in one simulator chamber in proximal contact, similar to the oral situation with the two restored proximal parts in a normal intercuspidation [15]. The two adjacent lateral ridges were occluded against a steatite (a multi-component semi-porous crystalline ceramic material) antagonist (6 mm in diameter) for 100,000 cycles at 50 N at a frequency of 0.5 Hz. The specimens were previously subjected to 2500 thermal cycles between + 5 and + 55 °C (THE 1100, SD Mechatronic, Munich, Germany). The mechanical action and the water temperature were checked periodically to ensure a reliable thermo-mechanical loading (TML) effect. After completion of TML, a second set of replicas was manufactured for later SEM analysis.

The replicas were mounted on aluminum stubs, sputter-coated with gold, and examined under a SEM (Phenom, FEI, Amsterdam, The Netherlands) as before at × 200 magnification. SEM examination was performed by one operator having experience with quantitative margin analysis who was blinded to the restorative procedures. The marginal integrity between resin composite and dentin was expressed as a percentage of the entire margin length in enamel and dentin. Marginal qualities were classified according to the criteria “gap-free margin,” “gap/irregularity,” and “not judgeable/artefact” (Figs. 4, 5, 6, 7, 8, 9, and 10). Afterwards, the percentage “gap-free margin” in relation to the individual judgeable margin was calculated as marginal integrity.

Statistical analysis was performed using SPSS/PC+, Version 17 (SPSS Inc., Chicago, IL, USA) for Windows. As the majority of groups in each of the two investigations (i.e., enamel or dentin marginal integrity) did not exhibit normal data distribution (Kolmogorov-Smirnov test), non-parametric tests were used (Kruskal-Wallis test, Wilcoxon matched-pairs signed-rank test, Mann-Whitney *U* test) for pairwise comparisons at the 95% significance level regarding the variables “percentage of gap-free margins.”

### In vivo observation

Patients were treated routinely in a private practice and retrospectively observed during routine recalls. Due to its characteristic as observational study, the STROBE checklist was followed [33].

Selection criteria were (1) absence of pain from the tooth to be restored; [2] possible application of rubber dam during restoration; [3] absence of any active periodontal and pulpal disease in the restored quadrant; [4] age 18–65; and [5] no pregnancy.

**Fig. 3:**
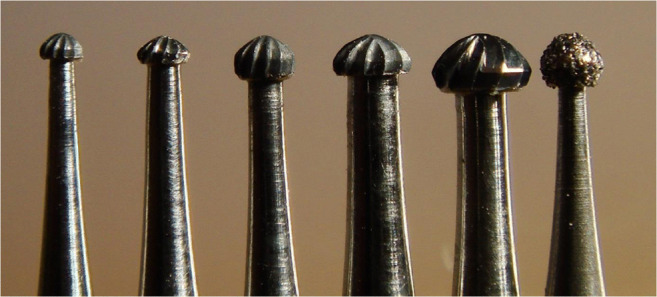
Different prototypes of mushroom-shaped burs for undermining and tunnel preparations

**Fig. 4:**
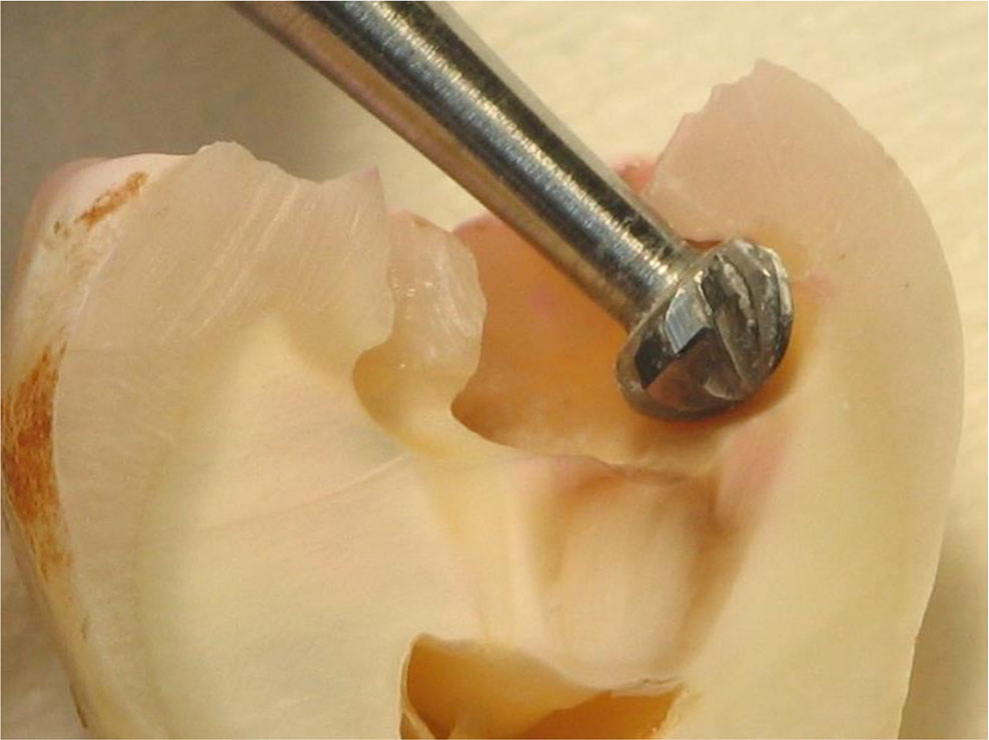
Mushroom-shaped bur for removal of undermining dentin caries

**Fig. 5:**
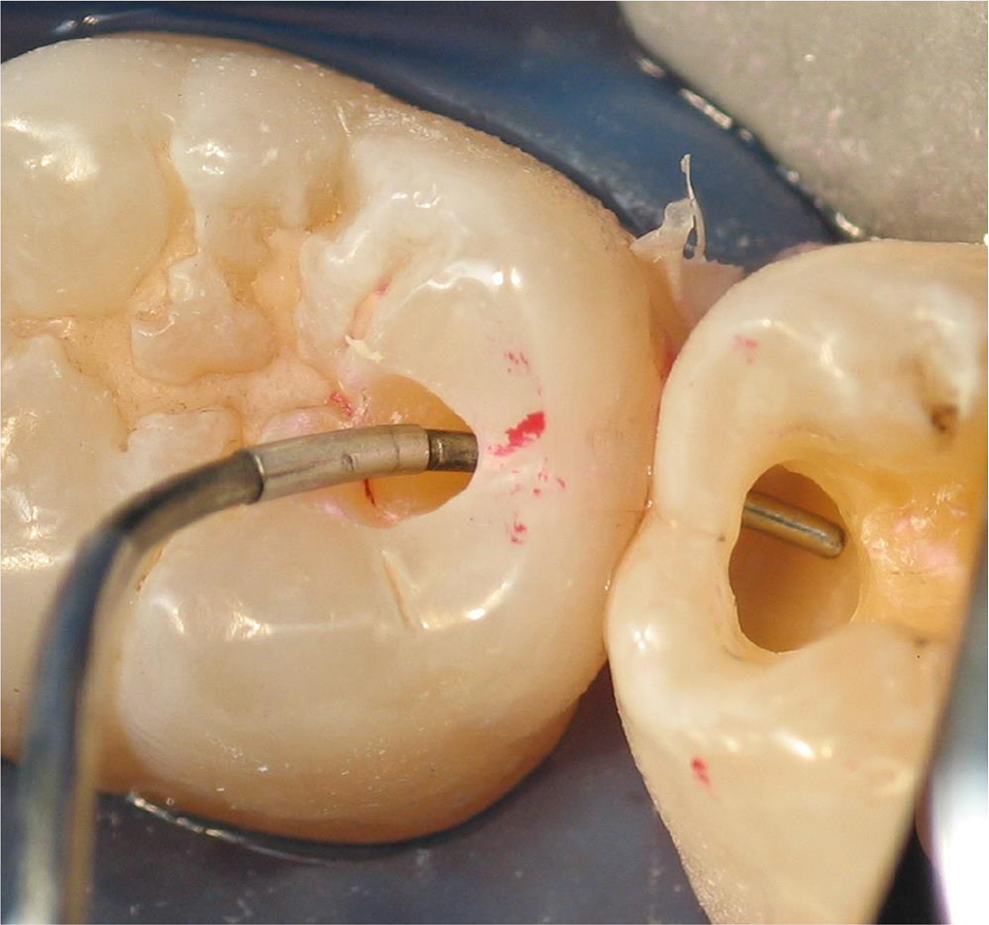
Two adjacent tunnel preparations in the clinical observation study. The right tunnel still provides a stable lateral ridge; the left tunnel provides a very stable lateral ridge

**Fig. 6:**
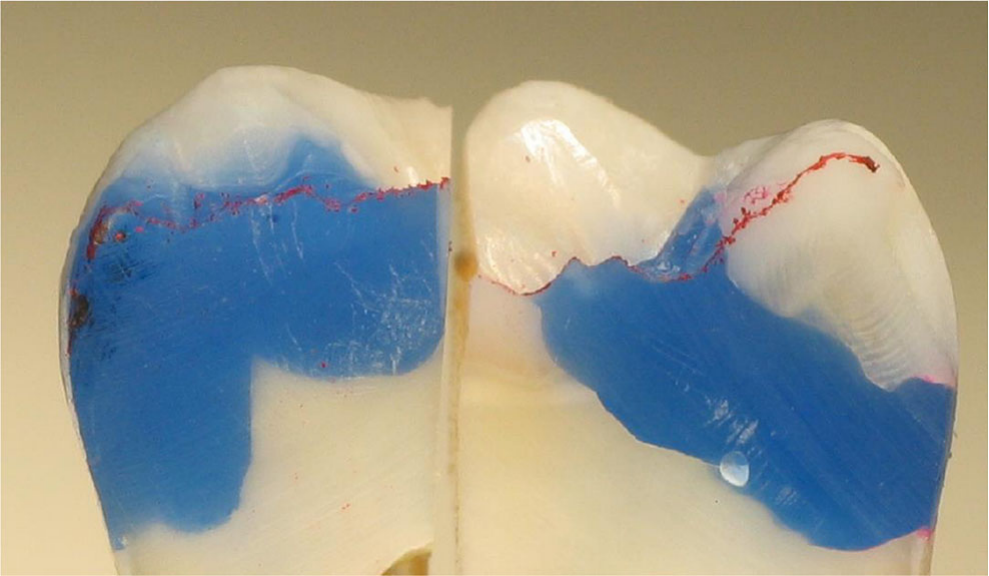
Clinically, box preparations (left) vs. tunnel preparations (right) were compared like in this cross-section filled with blue wax for better visibility

A total of 229 patients received 673 proximal resin composite restorations. From 371 tunnel restorations, 205 cavities were filled with resin composite (Amelogen Plus) without flowable composite lining (group tunnel A). A total of 166 tunnels were restored with additional use of a flowable composite (tunnel B, i.e., application of a thin (< 0.5 mm) layer of flowable composite (UltraSeal) which was light-cured prior to the application of the sculptable resin composite). A total of 302 teeth received conventional box-only preparations having been restored with sculptable composite only. For maximum protection of sound tooth hard tissues, a special mushroom-shaped bur was developed and prototyped as a carbide bur from 010 to 023 ISO size (Fig. 3). The new shape allowed for both extremely undermining excavation without removing substantial amounts of sound enamel, and conventional excavation (Fig. 4). For better protection of adjacent enamel in tunnel preparations, the anterior profile was designed flat (Fig. 3). Tunnel preparations were only considered when the mesiodistal width of the intact lateral ridge was > 2 mm (Fig. 5). In cases with near-to-complete or complete loss of the lateral ridge, a classical box-only preparation was cut. In a few cases, undermining caries toward buccal or oral aspects required extended access preparations and made tunnels impossible (Fig. 6).

**Fig. 7:**
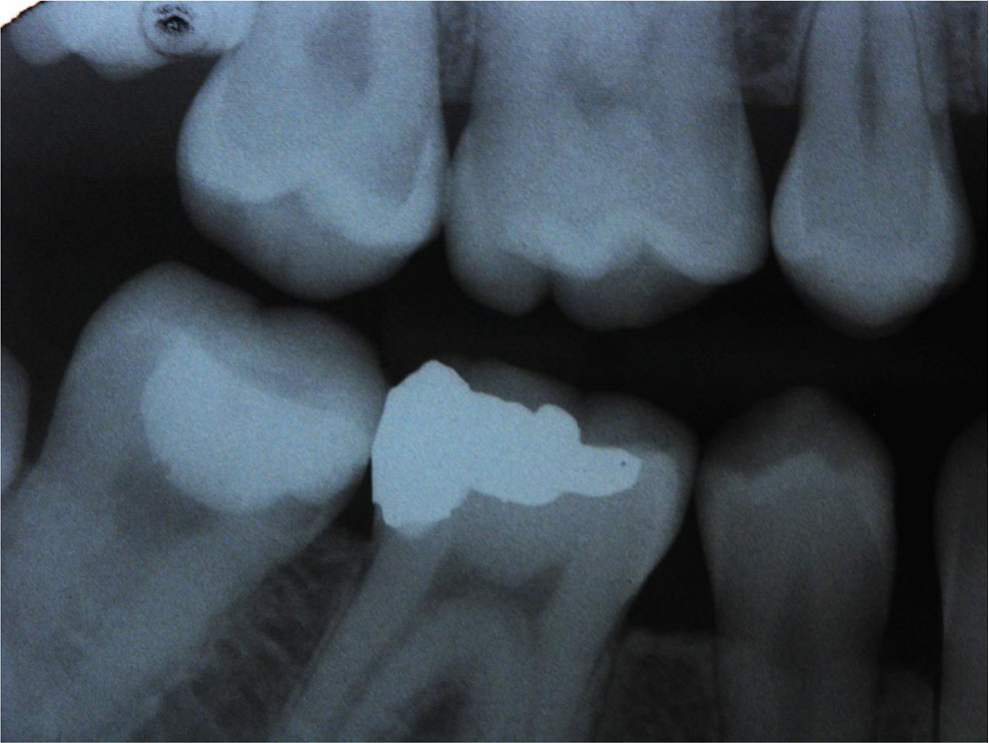
Radiograph showing caries mesially in the second upper molar

**Fig. 8:**
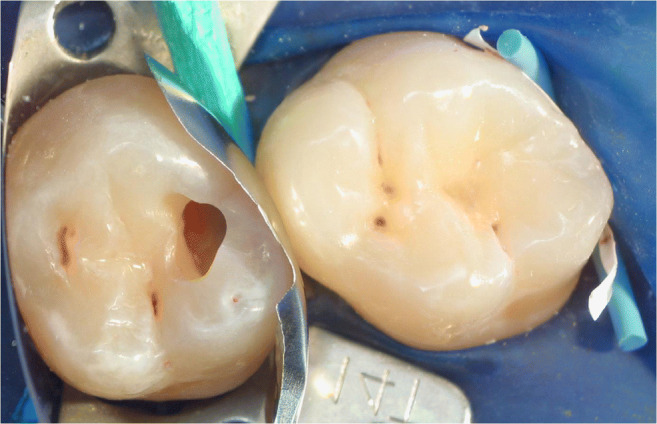
Clinical view corresponding to Fig. 8. Prior to restoration, a sectional matrix was placed and wedged

**Fig. 9:**
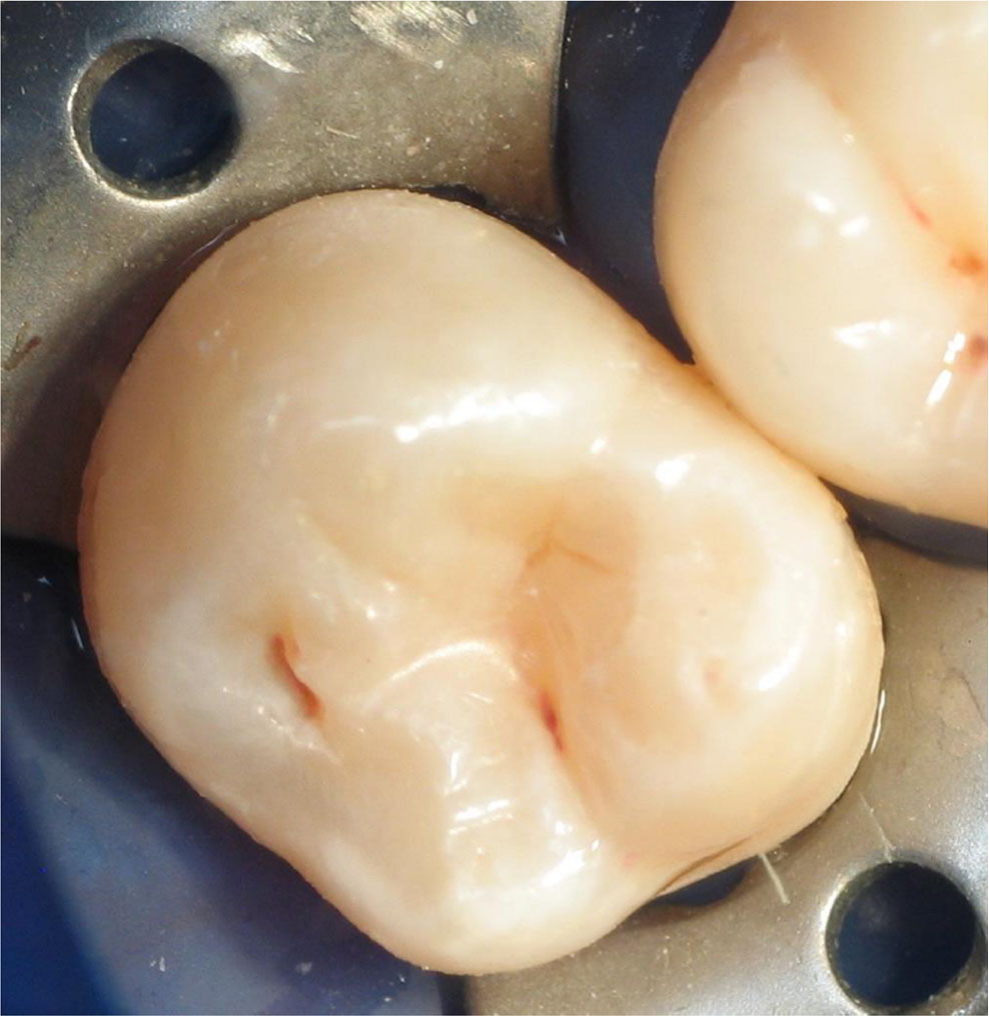
Finished restoration of Fig. 9

**Fig. 10:**
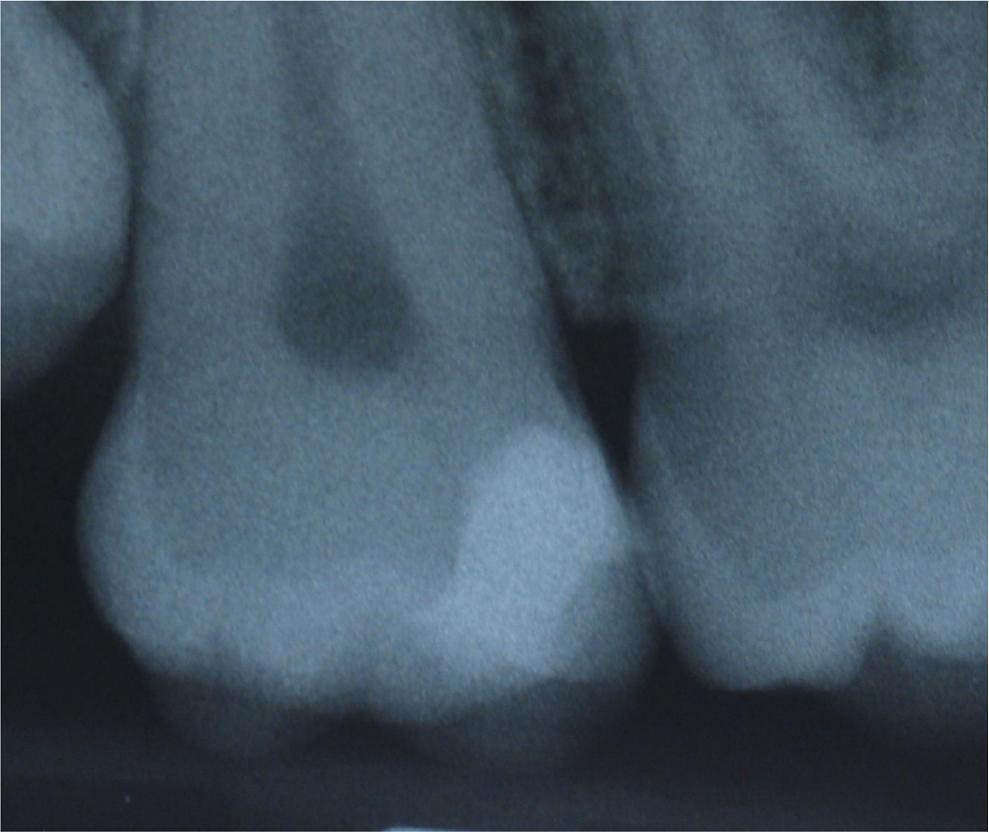
Corresponding radiograph of Figs. 8 and 9

**Fig. 11:**
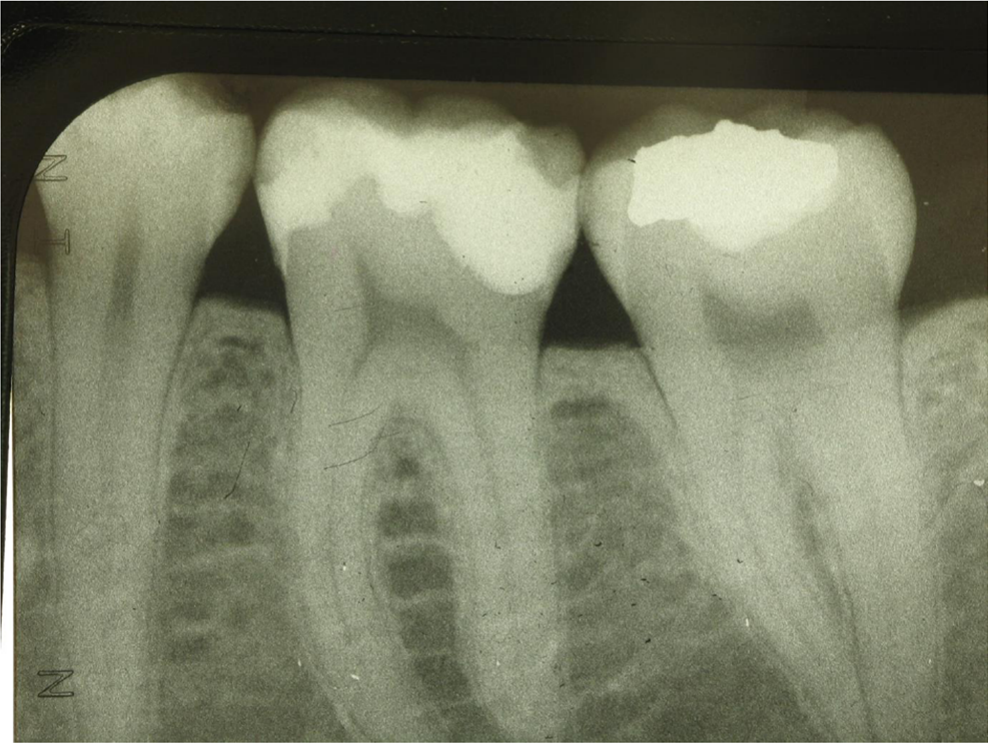
Tooth with two tunnel restorations after 5 years

Whereas 34% of lesions for box-restorations have been replacement of pre-existing restorations (amalgam or resin composite), in the tunnel groups, only primary lesions with no pre-existing proximal restoration have been applied. Nevertheless, it was attempted to balance the groups in terms of cavity volume and size in order to get similar conditions clinically (Fig. 7, 8, 9, 10, and 11). Preparations were performed under rubber dam and always under protection of adjacent teeth by applying an Inter-Guard (Ultradent). All restorative procedures were carried out using loups and coaxial LED light.

After applying and wedging a sectional matrix (Fig. 8), the complete cavity was etched with iBond Etch 35 (Kulzer) for 15 s and then rinsed for 15 s and carefully dried. After a rewetting step using one drop of tap water on a microbrush, adhesive pretreatment was performed using PQ1. Tunnel A was filled without lining with flowable composite; in tunnel B groups, proximal margins were sealed with a thin layer of a flowable composite (Ultra-Seal, Ultradent) having been light-cured separately. The main volume of the cavities was restored with the microhybrid composite Amelogen Plus in a meticulous layering technique. As soon as polymerization was completed, the surface of the restoration was controlled for defects and corrected when necessary. Visible overhangs were removed with a scaler and rubber dam was removed. Contacts in centric and eccentric occlusion were controlled with foils and adjusted with finishing diamonds, shaped with flexible disks, super-fine disks, and polishing brushes (Hawe Neos, Bioggio, Switzerland).

At the initial recall (baseline, i.e., within 2 weeks), and after one, two, and 5 years, restorations were assessed according to the modified United States Public Health Service (USPHS) criteria by the operating dentist using loups with × 3.5 magnification, mirrors, probes, and intraoral photographs. The investigator had 40 years of clinical experience and was trained and calibrated by the senior author by additional calibration sessions.

Statistical appraisal was computed with SPSS. Statistical unit was one tooth, differences between groups were evaluated using *t* test, and changes over time were calculated with the Friedman test (*p* = 0.05). For estimated survival, a Kaplan-Meier survival curve was computed.

## Results

### In vitro

The results of the in vitro part of the present study are displayed in Table 2. Statistically significant different performances among groups were evident already before storage and TML: Restorations of group tunnel A and box A revealed significantly more gaps and marginal deficiencies in the proximal part than tunnel B and box B restorations (*p* = 0.008; Table 2). Tunnel B and box B did not show significant differences (*p* = 0.076; Table 2); however, tunnel A exhibited more gaps compared to box A (*p* = 0.021; Table 2). After water storage and TML, these relations were the same but even more pronounced (*p* < 0.011; Table 2).

**Table 2:** Results of the in vitro SEM margin analysis.

Experimental in vitro group	% gap-free margin (SD) before TML	% gap-free margin (SD) after TML
Box only A	92.3 (9.3) ^B^	79.2 (8.9) ^B^
Box only B	100 ^A^	87.9 (7.4) ^A^
Tunnel A	86.6 (11.2) ^C^	70.3 (12.2) ^C^
Tunnel B	100 ^A^	90.3 (6.6) ^A^

Same superscript letters within columns mean *p* > 0.05. It is visible that in groups without flowable lining, also prior to thermomechanical loading (TML), defects were detectable under the SEM at × 200 magnification

### In vivo

**Fig. 12:**
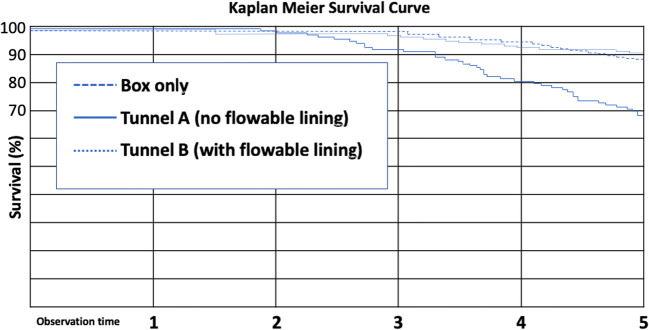
Kaplan-Meier survival curves for the three in vivo groups box-only, tunnel A, and tunnel B

In vivo, for observation group tunnel A, 138 out of 205 tunnels (67.3%) were evaluated over the whole period, retrospectively. Reasons for drop out were patients having been leaving practice or city (65%), further prosthodontic treatment (15%), and others (20%). A total of 69.4% of restorations were rated clinically acceptable (40% alpha, 29.4% bravo), and 30.6% of restorations had to be replaced due to wear (1.4%), marginal gap formation (13.0%), marginal staining (1%), marginal fractures (1.4%), secondary caries (2.2%), and lateral ridge fractures (11.6%). The resulting annual failure rate was 6.1% (Fig. 12).

In observation group tunnel B, 61 out of 166 tunnels (36.7%) could be evaluated. Ninety-five percent of restorations were clinically acceptable (59% alpha, 36% bravo), and 9% of restorations had to be replaced due to gap formation (3%), marginal staining (3%), and fractures of lateral ridges (3%). The resulting overall annual failure rate was 1.8% (Fig. 12).

In the course of observation of box-only restorations, 135 out of 302 restorations (44.7%) could be evaluated. Eighty-nine percent of restorations were clinically acceptable (29% alpha, 60% bravo), and 11% of restorations had to be replaced due to marginal fractures, chippings, marginal staining, and secondary caries resulting in an overall annual failure rate of 2.2%.

Regarding annual failure rates, tunnel A performed inferior compared to the other groups (*p* < 0.05). The performance of tunnel B and box-only was similar for most of the evaluated clinical criteria (*p* > 0.05); however, tunnels exhibited a larger percentage of sufficient proximal contacts (*p* < 0.05; 98 vs. 84%), retrospectively.

## Discussion

The aim of the present study was to estimate the clinical potential of tunnel vs. box-only preparations for bonded resin composite restorations. Traditionally, the beneficial effects of proximal tunnel restorations such as proximal contour or reduced absolute margin length have been more or less neutralized by a more demanding operative technique and a higher risk of fractures of the previously saved sound lateral ridge [18–25]. However, many of these assumptions are derived from clinical observations with glass ionomer cements, but without the instrument of adhesive re-stabilization, sophisticated tunnel experiments should have no chance to really withstand occlusal forces in vivo [20, 21, 23].

The present study clearly indicates that using bonded resin composites, clinical management of tunnels is possible. However, it also turned out that an intermediate layer of flowable resin composite is able to dramatically improve results both in vitro and in vivo. Especially, the fact that significant differences in vitro occurred already prior to TML was a clear hint in favor of this particular theory. This means that of course, far less occlusal load combined with a significantly shorter margin length is advantageous in tunnel situations; however, tunnels are far more demanding for the operator’s skills and experience [26]. It is furthermore worth to be mentioned in the discussion that the involved operator had 40 years of clinical experience and handling of minimally invasive resin composite restorations. It may be not expected that far less skilled and trained operators would perform equal in this special discipline. Nevertheless, also with the present experience, only the additional use of a flowable lining really caused sufficient margins for tunnel restorations. Another critical point in the present methodological setup is the retrospective, observational character of the in vivo part with the operator and investigator being the same person. This was finally the reason why we chose the STROBE approach in clinical observations [33]. Finally, the rather high number of restorations makes the clinical part interesting, despite a rather high dropout rate over the observation period of 5 years.

Concerning preclinical and clinical performance of proximal resin composite restorations, a positive effect of flowable resin composites or filled adhesives has been widely discussed [34]. This was previously described as elastic cavity wall concept; however, it seems not to be logical that a rather stiff material like a flowable resin composite should really be able to act as a stress breaker [34, 35]. These doubts are also reflected by the fact that there are many different conclusions found in the literature [34, 35]. From the clinical point of view, it nevertheless seems to make sense to use flowable resin composites as lining when posterior cavities are restored adhesively. This may be less attributed to an elastic cavity wall but more to a better adaptation to cavity walls as well as appropriate polymerization of the interface in enamel and dentin as well as adequate filling of marginal bevels [36].

It could be demonstrated that the overall success rate of tunnel preparation compared to box-only cavities no longer justifies to sacrifice considerable amounts of healthy tooth hard tissues in order to get easier access to infected dentin. *Primum nihil nocere* is the primary goal of minimum intervention not only in dentistry.

Facing the in vitro results gives a different picture compared to previous studies involving TML [7–9]. In many other in vitro studies, pronounced fatigue phenomena of resin-tooth interfaces were observed, especially in dentin [7–9]. In the present setup, only enamel margins were observed in vitro, so this effect was far less. This means that the key factor in the present investigation was not primarily fatigue of tooth-biomaterial interfaces but more clinically related defects during a demanding placement technique under simulated clinical conditions. This was proven by the fact that characteristic observations have been already seen before TML.

The present findings support the ability of the innovative mushroom-style bur used in this study to meet the requirements of minimally invasive dentistry as well as of universal use for excavation. The mushroom bur truly facilitated both the preservation of sound tooth hard tissues and intracoronal reconstruction. Further improvement of tunnel performance may be expected with more and more reliable marginal seal. A previously described application technique using not separately cured flowable resin composite might produce even more promising results, as indicated in a case presentation [17]. Also, in the present retrospective investigation, tunnel B exhibited no recurrent caries over the 5-year observation period. Finally, the null hypotheses had to be rejected.
