# Correction to: Class II resin composite restorations—tunnel vs. box-only in vitro and in vivo

**DOI:** 10.1007/s00784-021-04101-5

**Published:** 2021-08-16

**Authors:** Peter J. Preusse, Julia Winter, Stefanie Amend, Matthias J. Roggendorf, Marie-Christine Dudek, Norbert Krämer, Roland Frankenberger

**Affiliations:** 1 Marburg, Germany; 2 Department of Operative Dentistry, Endodontics, and Pediatric Dentistry Medical Center for Dentistry, University Medical Center Giessen and Marburg, Campus Marburg, Georg-Voigt-Str. 3, 35039 Marburg, Germany; 3 Department of Pediatric Dentistry, Medical Center for Dentistry, University Medical Center Giessen and Marburg, Campus Giessen, Schlangenzahl 14, 35392 Giessen, Germany

**Correction to: Clinical Oral Investigations**

**https://doi.org/10.1007/s00784-020-03649-y**

The article “Class II resin composite restorations—tunnel vs. box-only in vitro and in vivo”, written by Peter J. Preusse, Julia Winter, Stefanie Amend, Matthias J. Roggendorf, Marie-Christine Dudek, Norbert Krämer and Roland Frankenberger, was originally published Online First without Open Access. After publication in volume 25, issue 2, page 737–744 the author decided to opt for Open Choice and to make the article an Open Access publication. Therefore, the copyright of the article has been changed to © The Author(s) 2020 and the article is forthwith distributed under the terms of the Creative Commons Attribution 4.0 International License, which permits use, sharing, adaptation, distribution and reproduction in any medium or format, as long as you give appropriate credit to the original author(s) and the source, provide a link to the Creative Commons license, and indicate if changes were made. The images or other third party material in this article are included in the article’s Creative Commons license, unless indicated otherwise in a credit line to the material. If material is not included in the article’s Creative Commons license and your intended use is not permitted by statutory regulation or exceeds the permitted use, you will need to obtain permission directly from the copyright holder. To view a copy of this license, visit http://creativecommons.org/licenses/by/4.0.

The original article has been corrected.

